# Endocrinologic Abnormalities Observed Among Total Joint Arthroplasty Patients Using “Artri King” and Related Over-the-Counter Supplements: A Cautionary Tale from a Safety Net Hospital

**DOI:** 10.3390/jcm13237240

**Published:** 2024-11-28

**Authors:** McKenzie Culler, Cory K. Mayfield, Arjun Aron, Laura Del Val, Donald Longjohn, Nathanael D. Heckmann

**Affiliations:** 1 Department of Orthopaedic Surgery, Keck School of Medicine of the University of Southern California, Los Angeles, CA 90333, USA; mckenzie.culler@gmail.com (M.C.); cory.mayfield@med.usc.edu (C.K.M.); arjunaron@gmail.com (A.A.); longjohn@med.usc.edu (D.L.); 2 Rancho Los Amigos National Rehabilitation Center, Downey, CA 90242, USA; ldelval@dhs.lacounty.gov; 3 Rancho Research Institute, Rancho Los Amigos National Rehabilitation Center, Downey, CA 90242, USA

**Keywords:** Artri King, osteoarthritis, supplement, adrenal insufficiency

## Abstract

**Background/Objectives**: Artri King is an over-the-counter supplement previously marketed to treat joint pain and arthritis. In 2022, the Food and Drug Administration (FDA) issued a product warning after it discovered Artri King contained hidden ingredients including dexamethasone, diclofenac, and methocarbamol. Given the risk of adrenal insufficiency in the context of long-term dexamethasone use, we sought to report on adverse endocrinologic outcomes observed among patients endorsing the use of these supplements who presented to an orthopedic surgery clinic at a county safety net hospital. **Methods**: Preoperative patients presenting from November 2023 to June 2024 were screened for supplement use. Data were collected including patient demographics and comorbidities. Serum cortisol, adrenocorticotropic hormone (ACTH), and C-reactive protein (CRP) were obtained to assess adrenal function. Recommendations from Endocrinology regarding perioperative stress dose corticosteroids were also recorded. Standard descriptive statistics were employed to report our results. **Results**: In total, 13 patients (6 female and 7 male) were identified with a mean age of 62.8 years (range of 47–79 years) and an average BMI of 32.03 kg/m^2^ (range of 22.93–45.81 kg/m^2^). The average duration of use was 10.2 months (range of 1–36 months). One patient developed new-onset diabetes mellitus while taking supplements. Nine patients had low cortisol levels (<6.7 mcg/dL), necessitating referral to Endocrinology, and two were found to have concomitantly low ACTH levels (<5 pg/mL). Endocrinology recommended perioperative stress dose corticosteroids for all nine patients with low cortisol. **Conclusions**: Artri King and similar supplements may lead to severe endocrinological consequences. We recommend routine screening and continued management of patients who endorse supplement use.

## 1. Introduction

Osteoarthritis (OA) is the most prevalent form of arthritis, affecting roughly 595 million people worldwide, or 7.6% of the global population [1]. The burden of disease is expected to grow significantly from 2020 to 2050, particularly in the lower extremities, with OA cases in the knee anticipated to increase by 74.9% and cases in the hip to increase by 78.6% [1]. Chronic pain, the hallmark symptom of OA, can lead to loss of functional status, disability, and decreased quality of life in this patient population [2,3]. While current clinical practice guidelines endorse the use of non-steroidal anti-inflammatory drugs (NSAIDs) as a non-surgical therapeutic strategy for OA pain management, nearly 70% of patients with OA report using some form of supplement for their condition [4]. Furthermore, the true rate of use is likely higher, as patients generally tend to underreport their supplement use to healthcare providers in the clinical setting [5,6]. The use of unregulated supplements may lead to adverse health outcomes [7].

Artri King is an over-the-counter supplement previously marketed to treat joint pain and arthritis and sold widely at markets, pharmacies, and large retailers including Amazon and Walmart. In April 2022, the United States Food and Drug Administration (FDA) issued a warning on Artri King and similar supplement products due to initial reports of adverse health events including liver toxicity and death associated with their use [8]. Subsequent laboratory analysis revealed that Artri King contained hidden quantities of ingredients, such as dexamethasone, diclofenac sodium, and methocarbamol, that were not disclosed on product labels. The FDA issued a second warning in July 2023 after receiving reports that more than 30 additional consumers had experienced adverse outcomes including sudden weight gain, gastrointestinal bleeding, hyperglycemia, and adrenal dysfunction while using these supplements [8]. Following these warnings, manufacturers rebranded many of the supplements named by the FDA and continue to produce and distribute them without disclosing their hidden ingredients. Though major American retailers no longer sell Artri King or related products, patients are still able to purchase these supplements internationally in Mexico and Central America, as these countries do not enforce FDA warnings. Additionally, patients in the United States have reported acquiring these supplements domestically at swap meets, local markets, and online via eBay and other websites.

Dexamethasone and other corticosteroids exert a negative feedback effect on the body’s hypothalamic–pituitary–adrenal axis and decrease its endogenous production of cortisol, a regulatory steroid hormone [9,10]. As such, the use of corticosteroid-containing supplements may lead to adrenal dysfunction or life-threatening adrenal crisis [9,10]. The purpose of this study was to report on adverse endocrinologic outcomes observed among patients endorsing the use of Artri King and related supplements for pain associated with symptomatic osteoarthritis.

## 2. Materials and Methods

Preoperative patients presenting to a total joint arthroplasty clinic from November 2023 to May 2024 were screened by Orthopaedic clinicians during encounters for the use of Artri King and related supplements, including Artri Ajo King, Ortiga Mas Ajo Rey, and other products named in the FDA warnings. For patients endorsing supplement use, the length of use, dosage, and source of supplement acquisition were recorded. Patient demographic information including age, race and ethnicity, body mass index (BMI), and medical comorbidities were also recorded.

In response to FDA warnings and reports of adrenal dysfunction, patients disclosing supplement use were referred for same-day laboratory testing to evaluate for adrenal insufficiency. Laboratory tests measuring serum cortisol, adrenocorticotropic hormone (ACTH), and C-reactive protein (CRP) were ordered as recommended by Endocrinology specialists at our institution. Patients were instructed to present to the laboratory at the conclusion of the clinical encounter for testing. Patients whose tests yielded abnormal values consistent with adrenal dysfunction were referred to Endocrinology for further evaluation. Additionally, recommendations from Endocrinology regarding the need for perioperative and postoperative stress dose corticosteroids for total joint arthroplasty surgery were solicited and recorded by Orthopaedic clinicians.

Standard descriptive statistics were employed to report our results. Means and ranges were calculated for demographic characteristics including age, BMI, and duration of supplement use. Other demographic characteristics and endocrinologic findings were reported as frequencies. Correlations between the duration of supplement use and serum laboratory values were assessed using Spearman’s rank-order correlation. All statistical analyses were performed using RStudio (Version 2024.04.0+735, PBC, Boston, MA, USA).

## 3. Results

### 3.1. Patient Demographics

In total, 13 patients reported regularly taking Artri King and related supplements and were referred for laboratory testing (Table 1). 

The mean age was 63 (range of 47–79 years) and the majority of patients were men (7, 53.8%). All patients were Hispanic or Latino in origin and reported purchasing these supplements outside of the United States. The mean BMI was 32.03 kg/m^2^ (range of 22.93–45.81 kg/m^2^), with 7 patients reporting an increase in weight from baseline while taking Artri King. Seven patients reported a history of hypertension prior to taking Artri King. Nine patients were previously diagnosed with prediabetes and one patient was previously diagnosed with diabetes mellitus (Table 2). The average duration of supplement use was 10.2 months (range of 1–36 months).

### 3.2. Endocrinologic Findings

Of the 13 patients endorsing supplement use, 11 underwent same- or next-day serum laboratory testing. Two patients were temporarily lost to follow-up, one for six months and another for two months, before returning to the laboratory for the recommended tests. Nine patients (69.2%) were found to have low serum cortisol levels (<6.7 mcg/dL) by our institutional standards, necessitating a referral to Endocrinology for evaluation of adrenal insufficiency. Among these nine patients, two were found to have concomitantly low ACTH levels (<5 pg/mL). Serum CRP levels were elevated (>4.9 mg/L) in 6 of 13 patients (46.2%). One patient developed new-onset diabetes mellitus while taking Artri King and another patient developed new-onset prediabetes (Table 3). We found no significant correlation between the length of supplement use and serum cortisol (r = −0.045, *p* = 0.89), ACTH (r = −0.159, *p* = 0.60), or CRP (r = 0.520, *p* = 0.12). Endocrinology specialists recommended perioperative and postoperative stress dose corticosteroids with continued follow-up for all 9 patients with low cortisol levels.

## 4. Discussion

Artri King and related supplements contain undisclosed amounts of dexamethasone, a long-acting systemic corticosteroid. Corticosteroids reduce pain and inflammation by inhibiting the expression of proinflammatory cytokines such as IL-1b, IL-6, and tumor necrosis factor alpha (TNF-a) [11,12,13]. However, the unprescribed use of systemic corticosteroids is not recommended in the clinical setting of osteoarthritis, as these medications may induce disruption of the hypothalamic–pituitary–adrenal axis, leading to severe endocrinologic consequences. Despite FDA warnings about the potential dangers of these supplements, Artri King and similar products are still readily available for purchase through a number of avenues including local pharmacies, markets, and internationally. This series from our safety net orthopedic surgery clinic describes nine cases of adrenal insufficiency among preoperative patients taking corticosteroid-containing supplements with no other reported sources of exogenous steroid use. Our findings align with reports of Artri King-induced adrenal insufficiency and iatrogenic Cushing syndrome, a clinical syndrome resulting from adrenal suppression by exogenous glucocorticoids, that have been increasingly documented in the medical literature in recent years [14,15,16,17].

Prolonged use of systemic corticosteroids is associated with a multitude of adverse health outcomes including immunosuppression, osteonecrosis, new-onset diabetes mellitus, and cardiovascular disease [18,19]. Most importantly, as corticosteroids decrease endogenous cortisol production via suppression of the hypothalamic–pituitary–adrenal axis, rapid cessation of glucocorticoids can result in a life-threatening adrenal crisis [20]. Physiologic stressors such as infection, trauma, and surgery can also precipitate adrenal crises in these patients if the body is unable to respond appropriately by increasing endogenous cortisol production [20]. Current clinical guidelines indicate the need for perioperative and postoperative stress dose corticosteroid therapy for patients with adrenal insufficiency who undergo surgery in order to decrease the risk of adrenal crisis [21]. In our cohort, Endocrinology recommended perioperative and postoperative stress-dose corticosteroids for all nine patients who were found to have adrenal insufficiency associated with their use of these supplements prior to total joint arthroplasty.

Though we did not identify a correlation between length of supplement use and serum endocrinologic biomarkers, our patient data suggest that the adverse endocrinologic effects of corticosteroid-containing supplements may manifest within brief periods of use. Our patient who endorsed using Artri King on a daily basis for only one month was found to have a serum cortisol of 0.3 mcg/dL, well below the threshold for referral to Endocrinology for further evaluation. This suggests that regular use of these supplements, even for just a few weeks, may decrease the production of endogenous cortisol. Notably, two of the four patients with normal serum cortisol levels were temporarily lost to follow-up and returned to the laboratory several months after screening and cessation of supplement use.

In addition to the endocrinologic abnormalities observed and attributed to dexamethasone, the physiologic effects of other hidden ingredients in these supplements should also be explored. Diclofenac sodium, a non-steroidal anti-inflammatory drug (NSAID), reduces pain by inhibiting the enzyme cyclooxygenase (COX) and decreasing the production of prostaglandins [22,23]. Though generally well tolerated compared to other NSAIDs, diclofenac is associated with gastrointestinal side effects from decreased prostaglandin production, including gastritis and gastric ulcers [22]. Additionally, long-term use of NSAIDs may lead to severe renal damage, hematologic complications, or liver injury [22]. Methocarbamol, another hidden ingredient, is a centrally acting skeletal muscle relaxant that may be used to manage musculoskeletal pain, including pain associated with osteoarthritis, through an unknown mechanism of action [24,25]. It has been associated with a number of adverse neurologic and gastrointestinal effects, such as dizziness, falls, seizures, sedation, and gastritis [24,25]. Furthermore, patients using other medications while taking these supplements may be vulnerable to harmful drug–drug interactions. While much attention has been paid to the endocrinologic effects of these supplements due to the risk of dexamethasone-associated adrenal crisis, the potential of other hidden ingredients to cause harm or injury to patients, either directly or through interactions with other medications, must also be considered.

This study has several noteworthy limitations that must be addressed. First, the current case series does not have a comparator group and, as such, the findings outlined should be considered within the context of our descriptive analysis. Second, the small sample size makes it difficult to perform a robust analysis using inferential statistics. Finally, while no patients endorsed the use of exogenous glucocorticoids, it is possible that other medications or unreported comorbidities may have contributed to the endocrinologic abnormalities observed. Despite these limitations, we feel that our observations of adrenal dysfunction in this series of patients taking corticosteroid-containing supplements are important. The physiologic consequences of supplements containing hidden ingredients such as corticosteroids and anti-inflammatories are concerning, particularly for patients who may have altered organ function or contraindications to these ingredients at baseline. Further studies are needed to better determine the association between these supplements and specific adverse health outcomes.

As our findings demonstrate, it is essential to screen preoperative patients for the use of Artri King and other corticosteroid-containing supplements in order to assess for possible adrenal insufficiency. Failure to identify these patients during preoperative screening could result in devastating consequences associated with the physiologic stress of surgery, including life-threatening adrenal crises. We recommend continued screening for the use of over-the-counter supplements and a thorough assessment of endocrine function for patients who report using Artri King or related supplements or who present with clinical signs of Cushing syndrome in the context of recent supplement use.

## Figures and Tables

**Table 1:** Study cohort (n = 13).

Case	Sex	Age	Ethnicity	BMI (kg/m^2^)	Duration of Daily Use	Serum Cortisol (mcg/dL)	Serum ACTH (pg/mL)	Serum CRP (mg/L)
1	M	69	Hispanic/Latino	34.07	1 month	0.3	8	0.5
2 ^1^	F	71	Hispanic/Latino	26.88	3 months	12.2	20	Not obtained ^4^
3	M	57	Hispanic/Latino	26.71	4 months	3.3	40	0.4
4	M	61	Hispanic/Latino	33.86	5 months	5.2	25	19.1
5 ^2^	F	52	Hispanic/Latino	37.56	5 months	13	31	4.6
6	M	68	Hispanic/Latino	30.74	6 months	0.2	4	3.4
7 ^3^	F	79	Hispanic/Latino	22.93	6 months	5.8	35	Not obtained ^4^
8	M	47	Hispanic/Latino	45.81	6 months	0.4	6	9.2
9	M	63	Hispanic/Latino	26.22	12 months	0.9	6	Not obtained ^4^
10	F	69	Hispanic/Latino	35.58	12 months	9.2	61	11.4
11	F	57	Hispanic/Latino	35.16	12 months	0.4	4	5.1
12	F	64	Hispanic/Latino	32.73	24 months	7.2	13	9.5
13	M	62	Hispanic/Latino	28.08	36 months	2.5	24	8.3

^1^ Case 2 was lost to follow-up for six months after reporting supplement use. She presented to the laboratory six months later, at which point the serum laboratories listed were obtained. ^2^ Case 5 endorsed supplement use for a total of five months; she reported using the supplement daily for three months, then only twice monthly during months four and five. ^3^ Case 7 was lost to follow-up for two months after reporting supplement use. She presented to the laboratory two months later, at which point the serum laboratories listed were obtained. ^4^ Cases 2, 7, and 9 did not complete serum CRP testing for undisclosed reasons.

**Table 2:** Demographic characteristics of study cohort (n = 13).

Age, years (mean, range)	63	47–79
Sex (n, %)		
Women	6	46.2%
Men	7	53.8%
Race/Ethnicity (n, %)		
Hispanic/Latino	13	100%
Medical Comorbidities (n, %)		
Hypertension	7	53.8%
Prediabetes	8	61.5%
Diabetes Mellitus	1	7.7%
Body Mass Index, kg/m^2^ (mean, range)	32.03	22.93–45.81
Duration of Supplement Use, Months (mean, range)	10.2	1–36

**Table 3:** Summary of endocrinologic findings.

Low Serum Cortisol (<6.7 mcg/dL) (n, %)	9	69.2%
Low Serum ACTH (<5 pg/mL) (n, %)	2	15.4%
Elevated Serum CRP (>4.9 mg/L) (n, %)	6	46.2%
New-onset Diabetes Mellitus (n, %)	1	7.7%
New-onset Prediabetes (n, %)	1	7.7%

## Data Availability

The data presented in this study are available on request from the corresponding author because the data, including patient identifiers, are protected by HIPAA.
